# Extensive Phenotype of Human Inflammatory Monocyte-Derived Dendritic Cells

**DOI:** 10.3390/cells10071663

**Published:** 2021-07-02

**Authors:** Frédéric Coutant, Jean-Jacques Pin, Pierre Miossec

**Affiliations:** 1 Immunogenomics and Inflammation Research Team, University of Lyon, Edouard Herriot Hospital, 69437 Lyon, France; frederic.coutant@univ-lyon1.fr; 2 Immunology Department, Lyon-Sud Hospital, Hospices Civils de Lyon, 69310 Pierre-Bénite, France; 3 Eurobio Scientific/Dendritics—Edouard Herriot Hospital, 69437 Lyon, France; jean_jacques_pin@hotmail.fr; 4 Department of Immunology and Rheumatology, Edouard Herriot Hospital, 69437 Lyon, France

**Keywords:** dendritic cells, rheumatoid arthritis, C-type lectin receptors, DC-LAMP, Toll-like receptors

## Abstract

Inflammatory monocyte-derived dendritic cells (Mo-DCs) have been described in several chronic inflammatory disorders, such as rheumatoid arthritis (RA), and are suspected to play a detrimental role by fueling inflammation and skewing adaptive immune responses. However, the characterization of their phenotype is still limited, as well as the comprehension of the factors that govern their differentiation. Here, we show that inflammatory Mo-DCs generated in vitro expressed a large and atypical panel of C-type lectin receptors, including isoforms of CD209 and CD206, CD303 and CD207, as well as intracellular proteins at their surfaces such as the lysosomal protein CD208. Combination of these markers allowed us to identify cells in the synovial fluid of RA patients with a close phenotype of inflammatory Mo-DCs generated in vitro. Finally, we found in coculture experiments that RA synoviocytes critically affected the phenotypic differentiation of monocytes into Mo-DCs, suggesting that the crosstalk between infiltrating monocytes and local mesenchymal cells is decisive for Mo-DCs generation.

## 1. Introduction

Several dendritic cell (DC) subsets have been defined by their ontogeny, phenotype, and transcriptional profile, and all these subsets of DCs have been identified with altered phenotypes and functions in several chronic inflammatory/autoimmune disorders [1,2]. In humans, blood DCs are categorized as CD303^+^, CD304^+^, CD123^+^, plasmacytoid DCs (pDCs), and conventional DCs (cDCs), the latter being divided into two subsets, the CD1c^+^ DCs and the CD141^+^ DCs [3]. These DC subsets exhibit distinct Toll-like receptor (TLR) profiles that transduce inflammatory signals and then modulate their functions [4]. For instance, pDCs selectively express endosomal Toll-like receptor (TLR)7 and TLR9, which sense viral nucleic acids. Similarly to TLR, different DC subsets exhibit distinct C-type lectin receptors (CLRs), a family of surface receptors known to sense microbial carbohydrate moieties as well as products from dying cells [5,6]. The CD303 is specifically found at the surface of pDCs, whereas CD207 (Langerin) is expressed by a DC subset found in the skin, the Langerhans cells [7,8].

More recently, a third subset of DCs, named monocyte-derived DCs (Mo-DCs), has been described in patients with rheumatoid arthritis (RA) and in other inflammatory contexts, as well as in steady-state mucosal tissues [9,10,11,12,13]. These cells differentiate from monocytes recruited in inflamed tissues, and depending on the inflammatory environment, they promote T helper 1 (Th1), Th17, or Th2 responses [10,14,15,16,17,18,19]. Inflammatory Mo-DCs found in the synovial fluid of patients with RA have a complex and distinct phenotype compared to the other subsets of DCs. Indeed, they share markers found on blood CD1c^+^ DCs, such as CD1c or FcεRI, but also markers of monocyte-derived DCs such as CD1a, and markers of monocytes/macrophages such as the CD206 (mannose receptor) and the CD14 [10,20]. Consistent with their ability to secrete IL-12p70 and IL-23, Mo-DCs from the synovial fluid of patients with RA are inducers of Th1 and Th17 polarization [10]. Although the factors that govern the differentiation of Mo-DCs are still largely unknown, activation of the aryl hydrocarbon receptor seems to be an essential step for driving monocyte differentiation toward Mo-DCs [21].

To further characterize the phenotype of inflammatory Mo-DCs, we took advantage of an in vitro model that yields Mo-DCs closely resembling those found in vivo (19). We found that Mo-DCs expressed an atypical panel of CLR, as well as intracellular proteins on their membrane surface. We validated this phenotype on cells of the synovial fluid of patients with RA. Finally, we have shown that this phenotype is greatly affected by RA synoviocytes, suggesting that the crosstalk between monocytes and local mesenchymal cells has a heavy impact on Mo-DCs differentiation.

## 2. Materials and Methods

### 2.1. Cell Isolation

Mononuclear cells from healthy donors were isolated from human peripheral blood by density gradient centrifugation using Uni-sep maxi tubes (Eurobio scientific, Les Ulis, France) at 400× *g* for 20 min and then by centrifugation on a 50% Percoll solution at 400× *g* for 20 min. Recovered monocytes were around 80–90% pure as assessed by CD14 labeling. RA synoviocytes were isolated as previously described [22] from synovial biopsies of RA patients undergoing joint surgery and who fulfilled the American College of Rheumatology criteria for RA. Synovial tissue was minced into small pieces and then adhered in 6-well plates in DMEM (Eurobio scientific, Les Ulis, France), supplemented with 10% fetal bovine serum (FBS; Life Technologies, Carlsbad, CA, USA), 2 mM l-glutamine, and 100 U/mL penicillin/streptomycin. Cells were used between passages 4 to 9. For each preparation of RA synoviocytes, the absence of contamination by immune cells was checked by stainings with anti-CD45, anti-CD1, anti-CD3, anti-CD19, and anti-CD14 antibodies. At passages 4 to 9, 99% of the cells were negative for all of these markers and expressed CD44.

The protocol was approved by the Ethic Committee of the Hospitals of Lyon for the protection of persons participating in biomedical research (AC-2016-2729).

### 2.2. Cell Preparation and Culture

To generate inflammatory Mo-DCs, monocytes (1 × 10^6^ cells/mL) were cultured for 7 days in an RPMI-Glutamax medium (Eurobio scientific), supplemented with 10% FBS, glutamine, and antibiotics (penicillin and streptomycin), and in the presence of 100 ng/mL M-CSF (R&D systems, Minneapolis, MN, USA), 40 ng/mL IL-4 (Dendritics, Lyon, France), 5 ng/mL TNFα (R&D systems), and 62 nM of 6-formylindolo(3,2-b)carbazole (FICZ) (Enzo Life Sciences, Farmingdale, NY, USA), as previously described (19). For all the conditions, cells were incubated for 5–7 days, and cytokines were added only at the beginning of the culture. Medium was not refreshed during the course of the culture. The cell viability at the end of the differentiation was measured by propidium iodide (BD Biosciences, Franklin Lakes, NJ, USA) staining and was around 90%.

For coculture experiments, RA synoviocytes were seeded overnight in 6-well plates at a density of 1 × 10^5^ cells/well. The next day, supernatants were discarded, and the monocytes (2 × 10^6^ cells/well) were added on synoviocytes in 3 mL of medium. Times of incubation and culture conditions including concentrations of M-CSF, IL-4, TNFα, and FICZ were the same for coculture experiments and culture of monocytes alone.

### 2.3. Flow Cytometry

For phenotypic analysis, cells were incubated for 15 min at room temperature with monoclonal antibodies diluted at 5 µg/mL in PBS-5% FCS, followed by two washes. The following monoclonal antibodies were used: anti-CD14-FITC (clone MφP9, BD Biosciences), anti-CD1c-AF488 (clone AD5–8E7, Miltenyi-Biotec, Bergisch-Gladbach, Germany), anti-CD1a-AF647 (clone 214A9, Dendritics), anti-CD40-AF647 (clone G28-5, Dendritics), anti-CD80-AF647 (clone Mab104, Dendritics), anti-CD208-AF647 (clone 109G3, Dendritics), anti-CD209 like-AF647 (clone 118A8, Dendritics), anti-CD206-AF647 (clone 122D2, Dendritics), anti-CD367-AF647 (clone 111F8, Dendritics), anti-CD207-AF647 (clone 808E10, Dendritics), anti-CD303-AF647 (clone 104C12, Dendritics), CD123-AF647 (clone 107D2, Dendritics), anti-CD304-AF647 (clone 211H6, Dendritics), anti-TLR2-AF647 (clone 1308F10, Dendritics), anti-TLR3-AF647 (clone 1213F10, Dendritics), anti-TLR7-AF647 (clone 66H3, Dendritics), anti-TLR8-AF647 (clone 112H7, Dendritics), and anti-FDFO3-AF647 (clone 36H2, Dendritics). The percentage of positive cells and the mean fluorescence intensity (MFI) were determined with a Navios cytometer (Beckman Coulter, Miami, FL, USA). Specific MFIs were calculated by subtracting the MFI obtained on cells after stainings with the irrelevant antibody Mab 13 (anti-Bet V1 antibody, Dendritics).

Cells from synovial fluids were double-labelled using an AF488 labelled anti-CD209-like antibody combined with AF647 labelled anti-CD208, anti-CD303, or anti-CD123 antibodies, all from Dendritics. For single stainings, the following antibodies were used: anti-CD11b-PE (clone D12, BD Biosciences, Franklin Lakes, NJ, USA) and anti-CD16-FITC (clone NKP15, BD Biosciences) antibodies. Before analysis, cells were stained with propidium iodide (BD Biosciences) according to the manufacturer’s instructions. Flow cytometry analysis was performed by excluding cell debris and dead cells based on scatter signals and propidium iodide fluorescence.

### 2.4. Statistical Analysis

All statistical analyses were carried out using Prism version 6 software (GraphPad software Inc., San Diego, CA, USA). Differences between groups were analyzed using the paired *t* test. A P value less than 0.05 was considered as statistically significant.

## 3. Results

### 3.1. Inflammatory Mo-DCs Express a Large and Atypical Panel of C-Type Lectin Receptors

The pattern of expression of CLR by Mo-DCs was evaluated on cells generated with an in vitro culture model recently described [21]. In this model, human monocytes were cultured for 5 to 7 days in the presence of a cocktail of differentiation that included TNFα, in order to mimic the inflammatory context; IL-4, to induce the expression of CD1a, which is expressed on ex vivo Mo-DCs; and M-CSF, an essential cytokine for the differentiation of Mo-DCs in vivo in mice. The cocktail of differentiation was also supplemented with FICZ, a selective agonist of the aryl hydrocarbon receptor [21].

**Figure 1:**
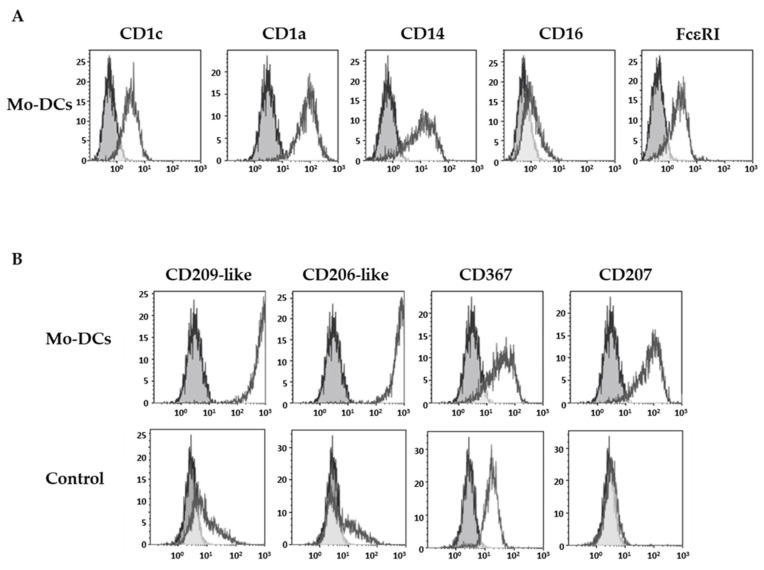
Inflammatory Mo-DCs display a large panel of C-type lectin receptors. Monocytes were cultured with the cocktail of differentiation (M-CSF, IL-4, TNFα, and FICZ) to generate Mo-DCs, or in the presence of 5 ng/mL TNFα (Control). At days 5–7, cells were stained with anti-CD1c, anti-CD1a, anti-CD14, anti-CD16, and anti-FcεRI antibodies (**A**) or with anti-CD209-like (DC-SIGN-like), anti-CD206-like (mannose receptor-like), anti-CD367 (DCIR), and anti-CD207 (Langerin) antibodies (**B**). Grey shaded histograms represent control stainings with an irrelevant antibody. Representative results of eight independent experiments for the condition Mo-DCs, and four independent experiments for the condition control cells.

As previously described [21], Mo-DCs expressed specific markers of DCs such as the CD1c and the CD1a, as well as CD14 and FcεRI, but they did not express the CD16 (Figure 1A).

The expression pattern of four CLRs was next investigated: the highly specific isoforms of the CD209 (CD209-like) and the CD206 (CD206-like), the CD367 (dendritic cell immunoreceptor), and the CD207 (Langerin). Stainings were performed with monoclonal antibodies obtained by immunization with human monocytes-derived DCs, which have been selected for their reactivity against isoforms mainly expressed by DCs, and their absence of reactivity against a panel of hematopoietic-derived cell types. As illustrated in Figure 1B, surface stainings of Mo-DCs with anti-CD209-like and anti-CD206-like antibodies were very high (average MFI value of 413 ± 59 and 406 ± 61, respectively, *n* = 5; Table 1).

In contrast, monocytes incubated with TNFα at the same concentration as in the cocktail, and phenotyped at the same time of incubation as Mo-DCs, did not express CD209-like nor CD206-like, or did so at very weak levels, compared to Mo-DCs (*p* < 0.0001). As shown in Figure 1B and in Table 1, Mo-DCs also expressed CD207 (average MFI value: 13 ± 3), albeit at a lower level. We also highlighted an expression of CD367 on the surface of Mo-DCs (average MFI value: 41 ± 9, Table 1). However, the anti-CD367 antibody used for the staining was less specific for DCs than the previous one and also stained monocytes cultured with TNFα alone (control condition, Figure 1B).

Thus, Mo-DCs expressed a large panel of CLRs, including the isoforms of CD209 and CD206 at high levels, which confirms that these markers are highly selective for monocyte-derived DCs since they are known to be expressed by DCs differentiated from monocytes with GM-CSF and IL-4, but not by pDCs nor cDCs [23,24].

### 3.2. Inflammatory Mo-DCs Share Markers of Plasmacytoid Dendritic Cells

**Figure 2:**
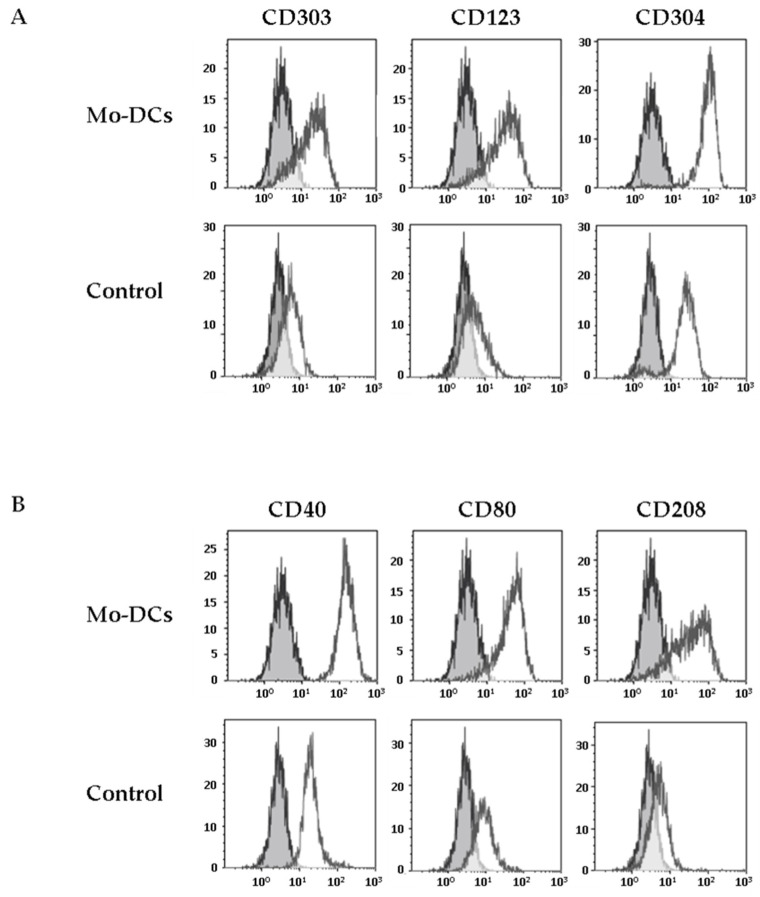
Inflammatory Mo-DCs share markers of plasmacytoid DCs and markers of activation. Monocytes were cultured with the cocktail of differentiation (Mo-DCs), or in the presence of TNFα (Control). Surface expression of plasmacytoid DCs markers (CD303, CD123, and CD304) was evaluated by flow cytometry (**A**), as well as the expression of costimulatory molecules (CD40, CD80) and the expression of the maturation marker CD208 (**B**). Grey shaded histograms are control stainings with an irrelevant antibody. Representative results of eight independent experiments for Mo-DCs, and four independent experiments for control cells.

We next explored the expression of CD303, another CLR considered as a specific marker of pDCs [7]. Strikingly, we found that Mo-DCs expressed low levels of CD303, unlike monocytes cultured in medium supplemented with TNFα (average MFI values: 7 ± 1 versus 1.6 ± 1.3, *p* = 0.01, Figure 2A and Table 1). The expression of CD303 by Mo-DCs generated in this culture model was confirmed by RT-PCR (Supplementary Figure S1A).

This unexpected result prompted us to evaluate the expression by Mo-DCs of other specific markers of pDCs, such as CD123 and CD304 (Neuropilin-1). As shown in Figure 2, Mo-DCs expressed both CD123 and CD304 (average MFI values: 16 ± 5 and 41 ± 6 respectively, Table 1). However, concerning the CD304, it is important to mention that this marker is less specific for pDCs than the others since it has been shown that CD304 is also expressed on the surface of Mo-DCs differentiated with GM-CSF and IL-4 [25].

In conclusion, Mo-DCs expressed a large and atypical panel of CLRs, combining high levels of the CLRs CD209-like and CD206-like, as well as CLRs found in other DC subsets, such as CD207 and CD303.

### 3.3. Inflammatory Mo-DCs Express the DC Maturation Marker CD208 at Their Surface

Mo-DCs are only found in inflammatory contexts. It is thus expected that these cells express costimulatory molecules such as CD40 or CD80 and the intracellular maturation marker CD208, a DCs lysosome-associated membrane glycoprotein (DC-LAMP), which is induced upon the process of DCs maturation [26]. We first evaluated the expression of costimulatory molecules at the surface of Mo-DCs. Mo-DCs expressed higher levels of CD40 and CD80 at their surface, compared to monocytes cultured in medium with TNFα (average CD40 MFI value: 139 ± 18 versus 8 ± 2, *p* < 0.0001; average CD80 MFI value: 16 ± 3 versus 4 ± 1, *p* = 0.01; Figure 2B and Table 1). Surprisingly, we found a positive staining of CD208 on the membrane surface of Mo-DCs, whereas CD208 was absent on the surface of monocytes cultured in medium with TNFα (average CD208 MFI value: 15 ± 4, Figure 2B and Table 1). We also confirmed CD208 gene expression by RT-PCR (Supplementary Figure S1A). To our knowledge, the only description of a positive staining of CD208 at the membrane surface of a cell was on human bronchioloalveolar carcinoma tumor cells, reinforcing the very unusual aspect of the phenotype of Mo-DCs [27].

### 3.4. Ectopic Expression of Intracellular TLRs by Inflammatory DCs

The identification of a surface expression of the intracellular protein CD208 led us to explore an ectopic localization of other intracellular proteins at the surface of Mo-DCs. Strikingly, low levels of the intracellular TLR7 (average MFI value: 8 ± 1, Table 1) were also detectable at the cell surface of Mo-DCs, as well as *tlr7* expression by RT-PCR (Supplementary Figure S1A,B). By contrast, surface stainings for the intracellular TLR3 and TLR8 were negative, as well as for TLR2 (Table 1).

### 3.5. Identification of CD209-Like^+^ CD208^+^ Cells in the Synovial Fluid of Patients with RA

**Figure 3:**
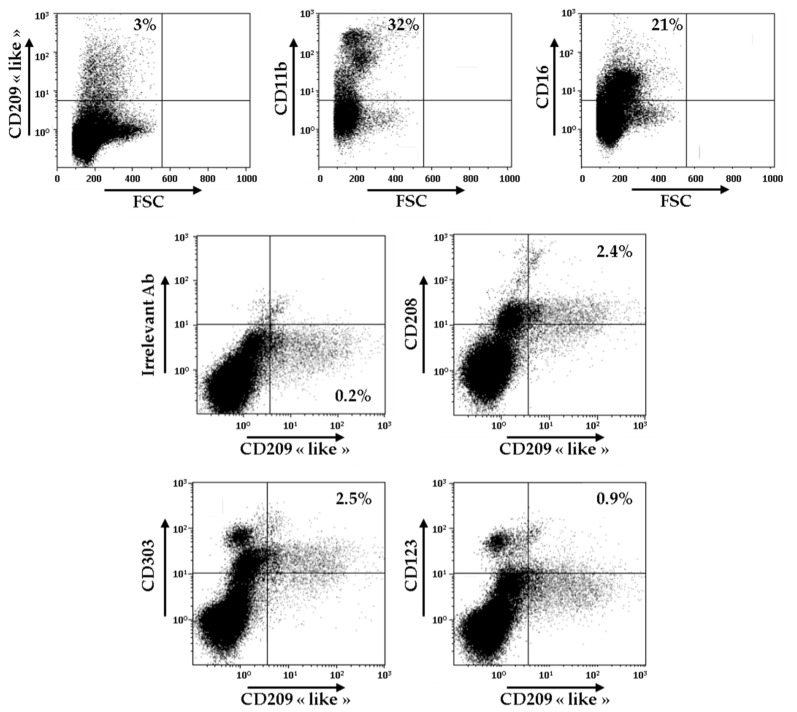
Identification of CD209-like^+^ CD208^+^ cells in the synovial fluid of patients with RA. Cells from one sample of RA synovial fluid were stained with anti-CD209-like, anti-CD11b, and anti-CD16 antibodies and analyzed by flow cytometry. Gating was performed on CD209-like positive cells, and cells were analyzed for the expressions of CD208, CD303, and CD123. Results are representative of two independent experiments.

In order to evaluate the relevance of these novel surface markers of Mo-DCs, we decided to carry out the analysis of the expression of CD209-like, CD208, CD303, and CD123 on the cells present in the synovial fluid of patients with RA. As shown in Figure 3, CD209-like positive cells represented around 3% of all the cells found in the synovial fluid, whereas cells CD11b^+^ and cells CD16^+^ constituted, respectively, 32% and 21% of all the cells.

As demonstrated by double staining experiments, CD209-like positive cells also expressed at their surface CD208, CD303, and, to a lower extent, CD123. These results indicate that the atypical combination of these markers is not only found on Mo-DCs generated in vitro, but exists also ex vivo, at least in the synovial fluid of patients with RA. However, since we performed a combination of double stainings, CD209-like/CD208, CD209-like/CD303, and CD209-like/CD123, we cannot rule out the possibility that several separate subsets of cells are present in the CD209-like+ cell population.

### 3.6. RA Synoviocytes Critically Affect the Differentiation of Monocytes into Mo-DCs

**Figure 4:**
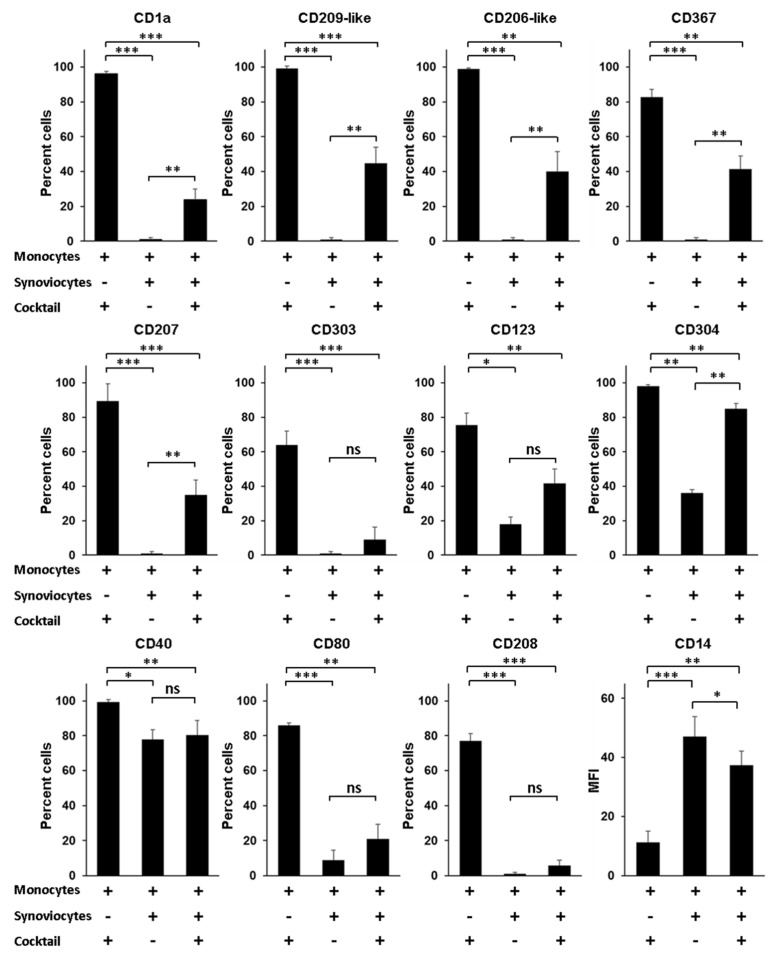
Rheumatoid arthritis synoviocytes impair the differentiation of monocytes into inflammatory dendritic cells. Monocytes were cultured in the presence or not of the cocktail of differentiation (M-CSF, TNFα, IL-4, and FICZ) for 5 days, with or without RA synoviocytes, and analyzed by flow cytometry at day 5. RA synoviocytes alone were negative for all the markers tested (data not shown). Results are represented as mean ± SEM of *n* = 4 independent experiments from 4 healthy donors (monocytes) and 3 RA patients (synoviocytes), except for CD123 and CD304 (*n* = 3). * *p* < 0.1, ** *p* < 0.01, *** *p* < 0.001 (Paired *t*-test).

A question still outstanding is the nature of the environmental factors that govern the differentiation of monocytes into Mo-DCs, especially when monocytes infiltrate the synovial tissue of patients with RA. Although Mo-DCs have been identified in the synovial fluid, no study has reported their presence in the synovial tissue. Yet, it seems reasonable to speculate that the process of differentiation from monocytes into Mo-DCs starts when monocytes exit blood and enter into the synovial tissue. In this scenario, interactions between infiltrating monocytes and local mesenchymal cells such as dysregulated synoviocytes could be a decisive trigger, able to induce in monocytes a program of differentiation into Mo-DCs. To evaluate this hypothesis, we first compared the phenotype of Mo-DCs generated in vitro after 5 days of culture in the presence of the cocktail of differentiation, to the phenotype of monocytes cocultured during 5 days with RA synoviocytes, without the cocktail. As shown in Figure 4, cells cocultured with RA synoviocytes were negative for specific Mo-DCs markers (CD1a, CD209-like, and CD206-like), as well as for the markers of pDC and Langerhans cells (CD303, CD123, CD304, and CD207).

Compared to Mo-DCs, the cells showed lower expression of the costimulatory molecules CD40 and CD80, and were negative for the expression of the DC maturation marker CD208. By contrast, the cells maintained high levels of expression of CD14 compared to Mo-DCs (47 ± 7 versus 11 ± 4, *p* = 0.0006). The phenotype of these cells cannot be explained by a possible toxicity linked to the coculture with RA synoviocytes since more than 90% of the cells were viable after PI staining (data not shown). These data indicated that RA synoviocytes did not support the differentiation of monocytes into Mo-DCs.

We next evaluated whether RA synoviocytes affect the differentiation of monocytes into Mo-DCs in the presence of the cocktail. After 5 days of culture, ≌40% or less of the cells generated in monocytes/RA synoviocytes cultures with the cocktail of differentiation had acquired the Mo-DCs markers (CD1a, CD209-like, and CD206-like), the CD367, and the markers of pDC and Langerhans cells (CD303, CD123, and CD207), although at lower levels than Mo-DCs (Figure 4). The percentages of the cells CD40^+^, CD80^+^, or CD208^+^ were similar to those observed on cells cocultured with RA synoviocytes in absence of the cocktail of differentiation, and the cells also expressed higher levels of CD14, compared to Mo-DCs (37 ± 5 versus 11 ± 4, *p* = 0.0012).

In conclusion, monocytes cocultured with RA synoviocytes in the presence of the cocktail of differentiation had a phenotype closer to monocytes than Mo-DCs, demonstrating that RA synoviocytes critically affected the differentiation of monocytes into Mo-DCs.

## 4. Discussion

The present study shows that several CLRs are expressed abundantly on the surface of Mo-DCs, such as CD209-like and CD206-like, the isoforms of CD209 and CD206, and to a lesser extent CD367, CD303, and CD207. This atypical combination of CLRs seems to be highly specific to Mo-DCs since pDCs, for instance, express solely CD303 but not CD209-like nor CD207, and Langerhans cells express CD207 but not CD303 [5,7,23]. Beyond the fact that this intriguing combination of markers could constitute a specific signature of Mo-DCs, it raises also the question of the biological role of these receptors. CLRs harbor a functional plasticity, some of them detecting endogenous molecules or other detecting molecules from infectious agents, and many of them act as dual receptors able to sense both endogenous and exogenous ligands [6]. This is the case, for example, for CD209, which binds various mannose- and fucose-containing viral, bacterial, fungal, and parasite-derived glycans, but also glycans that can be exposed on membrane receptors, such as ICAM-3 or ICAM-2 [28,29]. The consequences of the stimulation of CD209 are highly variable as they can promote the establishment of either Th1/Th17 or Th2-biased immune responses in distinct contexts [30,31,32]. For instance, in a murine model of *Schistosoma* infection, it has been recently shown that CD209 is necessary for the development of severe, Th17-mediated immunopathology [33]. Whether these CLRs are functional on Mo-DCs and what could be the consequences of a functional deregulation of these receptors in a chronic inflammatory context such as RA remain to be determined.

A second unexpected aspect that emerged from our phenotypic analysis of Mo-DCs was the identification of several intracellular proteins at the surface of Mo-DCs, such as CD208. Whether CD208 exert some specific functions when displayed at the cell surface has to be addressed. However, an attractive hypothesis would be that surface CD208 could be involved in the migration of Mo-DCs. Indeed, the only reported observation of surface CD208 was on tumor cells, and it has been shown that overexpression of CD208 was associated with increased metastatic potential through increased cell motility into lymph-vascular spaces [27]. Thus, by analogy with tumor cells, Mo-DCs expressing CD208 at their surface might migrate into the draining lymph nodes, or into ectopic lymphoid-like structures found in the synovial tissue of patients with RA [34]. These structures are characterized by aggregates of T and B cells and are suspected to be ectopic niches, which enable the activation of autoreactive T cells. Consistent with this hypothesis, mature DCs expressing CD208 have been observed in these structures [34]. Whether these cells also express other markers that we characterized on Mo-DCs such as CD209-like remains to be investigated. Whatever functions DC-LAMP might fulfill in Mo-DCs, the expression of this marker at the membrane surface provides an interesting opportunity for the characterization of Mo-DCs in vivo and/or for targeting strategies.

Finally, another key element of this study is the observation that RA synoviocytes critically affected the differentiation of Mo-DCs. The regulation of Mo-DCs differentiation in tissues remains elusive, as does the nature of the environmental factors in RA that govern their differentiation. We initially hypothesized that the program of monocyte differentiation into Mo-DCs should start when monocytes exit blood and enter into the synovial tissue. RA synoviocytes are characterized by somatic mutations and/or epigenetic changes that affect their functions [35]. The contact of monocytes with these dysregulated cells could then constitute a decisive trigger to induce within monocytes a program of differentiation into Mo-DCs. However, we demonstrated by coculture experiments that synoviocytes from patients with RA were not able to induce monocyte differentiation into Mo-DCs. Furthermore, we found that RA synoviocytes greatly affected the process of differentiation into Mo-DCs induced by the cocktail of cytokines. Several studies have reported the effects of stromal cells on the differentiation of monocytes towards DCs [36,37]. For instance, skin fibroblasts switch the differentiation of monocytes from DCs to macrophages [38]. However, the mechanisms that occur in monocytes cocultured with the RA synoviocytes in the presence of the cocktail of differentiation appear to be different from those that take place in coculture with skin fibroblasts. Indeed, in this condition and at the end of the incubation, cells were CD16-negative and seemed morphologically distinct from macrophages (data not shown). In contrast, these cells retained a positive labeling of CD14, suggesting that the differentiation of the monocytes appeared to be blocked by RA synoviocytes, despite the presence of the differentiation cocktail. It is important to emphasize that some of the cells at the end of the coculture expressed some markers found on Mo-DCs, such as the CD209-like. This suggests that an induction of the program of differentiation toward DCs might occur in monocytes that are in contact with RA synoviocytes, but other cellular or molecular factors are necessary for the generation of Mo-DCs expressing all the markers described in this study. However, we cannot formally exclude that RA synoviocytes affected the differentiation of Mo-DCs by consuming and/or neutralizing the cytokines necessary for this process. Experiments are underway to explore this possibility.

In conclusion, we have characterized the phenotype of Mo-DCs by using antibodies highly specific for DCs. The combination of markers identified on Mo-DCs generated in vitro allowed us to highlight a population of cells phenotypically close to the in vitro differentiated Mo-DCs in the synovial fluid of a patient with RA. Finally, we have demonstrated that stromal cells of RA synovial tissue play a critical role in the differentiation process of Mo-DCs. Although preliminary, these results could pave the way for therapeutic strategies targeting specifically Mo-DCs.

## Figures and Tables

**Table 1:** Phenotype of monocyte-derived inflammatory dendritic cells.

Antigen	Clone	MFI (SEM) ^1^
C-type lectin receptors		
CD209 isoform (DC-SIGN like)	118A8	413 (59)
CD206 (Mannose receptor)	122D2	406 (61)
CD367 (DCIR)	111F8	17 (4)
CD207 (Langerin)	808E10	13 (3)
CD303	104C12	7 (1)
CD304 (Neuropilin-1)	211H6	41 (6)
Toll-like receptors		
TLR2	1308F10	0
TLR3	1213F10	0
TLR7	66H3	8 (1)
TLR8	112H7	0
Antigen-presenting molecules		
CD1a	214A9	10 (2)
MHC-II	216H1	132 (23)
Costimulatory molecules/Maturation markers		
CD40	G28–5	139 (18)
CD80	Mab104	16 (3)
CD208 (DC-LAMP)	109G3	15 (4)
Interleukin receptor		
CD123	107D2	5 (2)
ITIM-bearing receptor		
FDFO3	36H2	28 (2)

^1^ Inflammatory dendritic cells at days 5–7 were stained with different antibodies listed above. Stained cells were analyzed by flow cytometry, and the MFI of each label was determined. Data represent the MFI ± SEM of 5 independent experiments. Abbreviations: DCIR, dendritic cell immunoreceptor; DC-LAMP, dendritic cell lysosomal associated membrane glycoprotein; DC-SIGN, dendritic cell-specific intercellular adhesion molecule-3-grabbing non-integrin; ITIM, immunoreceptor tyrosine-based inhibitory motif; MFI, mean fluorescence intensity.

## Data Availability

Correspondence and request for materials should be addressed to F.C., J.-J.P. or P.M.

## Supplementary Materials

The following are available online at https://www.mdpi.com/article/10.3390/cells10071663/s1, Figure S1: Expression of CD208, CD303 and TLR7 by inflammatory Mo-DCs.
