# Supplementary material for: Extensive Phenotype of Human Inflammatory Monocyte-Derived Dendritic Cells
Source: Cells. 2021 Jul 2;10(7):1663. doi: 10.3390/cells10071663 (PMC8307578; doi:10.3390/cells10071663)
Supplement: Supplementary file 1 [file cells-10-01663-s001.zip › cells-1258415-supplementary.pdf]

cells

MDPI

Supplemental information

Supplementary Materials and Methods

RNA extraction and real-time PCR

Total RNA of infDCs was extracted using the RNeasy Mini Kit (Qiagen®, Hilden, Germany) and quantified with the Quant-it kit assay (Invitrogen™ by Thermo Fisher Scientific, Grand Island, NY, USA) following the manufacturer's instructions. cDNA was synthesized using the QuantiTect reverse transcription kit (Qiagen®) according to the manufacturer's instructions. SYBR green-based real-time qRT-PCRs were performed on the CFX96 Real-Time PCR Detection System (BioRad, Hercules, CA, USA) using the QuantiFast SYBR green kit and QuantiTect primers (Qiagen®). Cycle threshold values for CD208, CD303,TLR7, and glyceraldehyde 3-phosphate dehydrogenase (GAPDH) genes were obtained from three independent Mo-DC RNA samples.

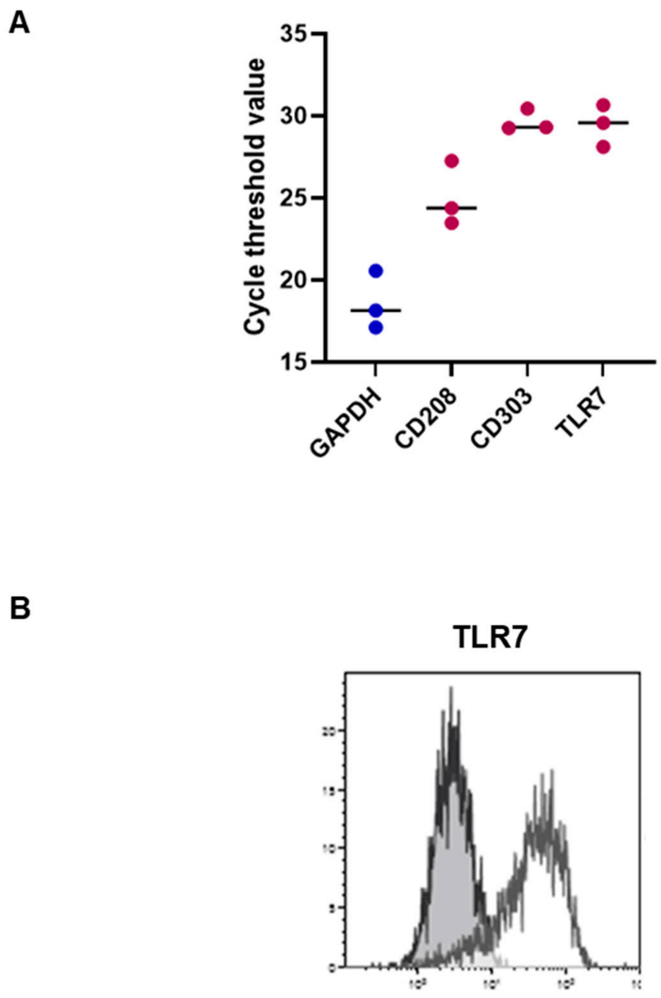

Supplementary Figure S1. Expression of CD208, CD303 and TLR7 by inflammatory Mo-DCs. (A) Real-time PCR were performed on monocytes cultured during 5 days in presence of the cocktail of differentiation (M-CSF, TNF, IL-4, FICZ). Cycle threshold values of GAPDH, CD208, CD303 and TLR7 are shown. Data presented are values of three independent experiments. (B) Surface expression of TLR7 on inflammatory Mo-DCs. Grey shaded histograms are control stainings with an irrelevant antibody. Representative results of five independent experiments.
